# Stride Length Predicts Adverse Clinical Events in Older Adults: A Systematic Review and Meta-Analysis

**DOI:** 10.3390/jcm10122670

**Published:** 2021-06-17

**Authors:** Ibadete Bytyçi, Michael Y Henein

**Affiliations:** 1 Institute of Public Health and Clinical Medicine, Umeå University, 90187 Umea, Sweden; i.bytyci@hotmail.com; 2 Clinic of Cardiology, University Clinical Centre of Kosovo, 10000 Prishtina, Kosovo; 3 Department of Nursing, Universi College, 10000 Bardhosh, Kosovo; 4 Molecular and Clinic Research Institute, St George University, London SW17 0QT, UK; 5 Institute of Fluid Dynamics, Brunel University, London UB8 3PH, UK

**Keywords:** stride length, adverse clinical events, older adult

## Abstract

Background: This meta-analysis aims to estimate the power of walking stride length as a predictor of adverse clinical events in older adults. Methods: We searched all electronic databases until April 2021 for studies reporting stride length and other spatial gait parameters, including stride velocity, stride width, step width and stride variability, and compared them with clinical outcomes in the elderly. Meta-analyses of odds ratios (ORs) of effects of stride length on clinical outcomes used the generic inverse variance method and random model effects. Clinical outcomes were major adverse events (MAEs), physical disability and mortality. Results: Eleven cohort studies with 14,167 patients (mean age 75.4 ± 5.6 years, 55.8% female) were included in the analysis. At 33.05 months follow up, 3839 (27%) patients had clinical adverse events. Baseline stride length was shorter, WMD −0.15 (−0.19 to −0.11, *p* < 0.001), and stride length variability was higher, WMD 0.67 (0.33 to 1.01, *p* < 0.001), in fallers compared to non-fallers. Other gait parameters were not different between the two groups (*p* > 0.05 for all). Short stride length predicted MAE OR 1.36 (95% CI; 1.19 to 1.55, *p* < 0.001), physical disability OR 1.26 (95% CI; 1.11 to 1.44, *p* = 0.004) and mortality OR 1.69 (95% CI; 1.41 to 2.02, *p* < 0.001). A baseline normalized stride length ≤ 0.64 m was more accurate in predicting adverse clinical events, with summary sensitivity 65% (58–71%), specificity 72% (69–75%) and accuracy 75.5% (74.2–76.7%) compared to stride length variability 5.7%, with summary sensitivity 66% (61–70%), specificity 56% (54–58%) and accuracy 57.1% (55.5–58.6%). Conclusion: The results of this meta-analyses support the significant value of stride length for predicting life-threatening clinical events in older adults. A short stride length of ≤0.64 m accurately predicted clinical events, over and above other gait measures.

## 1. Introduction

Advances in medical management of patients with various conditions, particularly cardiovascular conditions, have resulted in a significant increase in longevity [1,2]. This older population may, however, be limited by other medical problems, including arthro-skeletal stiffness and its consequences, e.g., physical disability and falls, which may lead to a decline in the functional capacity and quality of life as well as increased risk of dependence and institutionalization [3,4]. Physical disability also reflects difficulties that individuals may experience in interaction with society [5], which can lead to psychological disorders [6].

Physical activity/exercise is an essential disease-preventive measure, irrespective of age [7]. While standards and objective targets are well established, they might not necessarily apply to older people because of other various comorbidities or pre-existing chronic conditions [8]. Gait speed has been shown to be associated with better survival among older adults and to reflect health and functional status [9,10]. This study aims at assessing, in a meta-analysis format, the clinical relevance of gait measurements and their predictive value of falls, disabilities and even mortality in senior communities.

## 2. Methods

### 2.1. Study Design

This study was designed according to the guidelines of the 2009 preferred reporting items for systematic reviews and meta-analysis (PRISMA) statement and amendment to the Quality of Reporting of Meta-analyses (QUOROM) statement [11]. Because of the study design (meta-analysis), neither Institutional Review Board (IRB) approval nor patient informed consent was needed.

### 2.2. Search Strategy

Two reviewers searched all electronic databases (PubMed-Medline, EMBASE, Scopus, Google Scholar, the Cochrane Central Registry of Controlled Trials and ClinicalTrial.gov, accessed on 25 May 2021) until April 2021 using the key words “Stride length” OR “Stride variability” OR “spatial gait” AND “Clinical outcomes” OR “adverse clinical outcome” OR “Physical disability” OR “Mortality” OR “Dependency” OR “Institutionalization” OR “Falls” AND “Older adult population” OR “Older adult”. The wild-card term “*” was used to enhance the sensitivity of the search strategy. The literature search was limited to articles published in English and to human studies. No filters were applied during the search, and two reviewers (IB and MYH) independently evaluated each article. The remaining articles were obtained in full text and assessed by the same researchers.

### 2.3. Study Selection

Original studies were included if they met the following criteria: (a) investigate older adult population with baseline stride length, (b) report predictors of outcome, (c) enroll population of adults aged ≥ 65 years, and (d) have follow-up data. Exclusion criteria were (i) patients with physical and functional disability, (ii) insufficient statistical data to test predictive value, (iii) studies not in older adult population, (iv) no follow-up data, and (e) articles not published in English.

### 2.4. Clinical Outcome Measures

**Figure 1:**
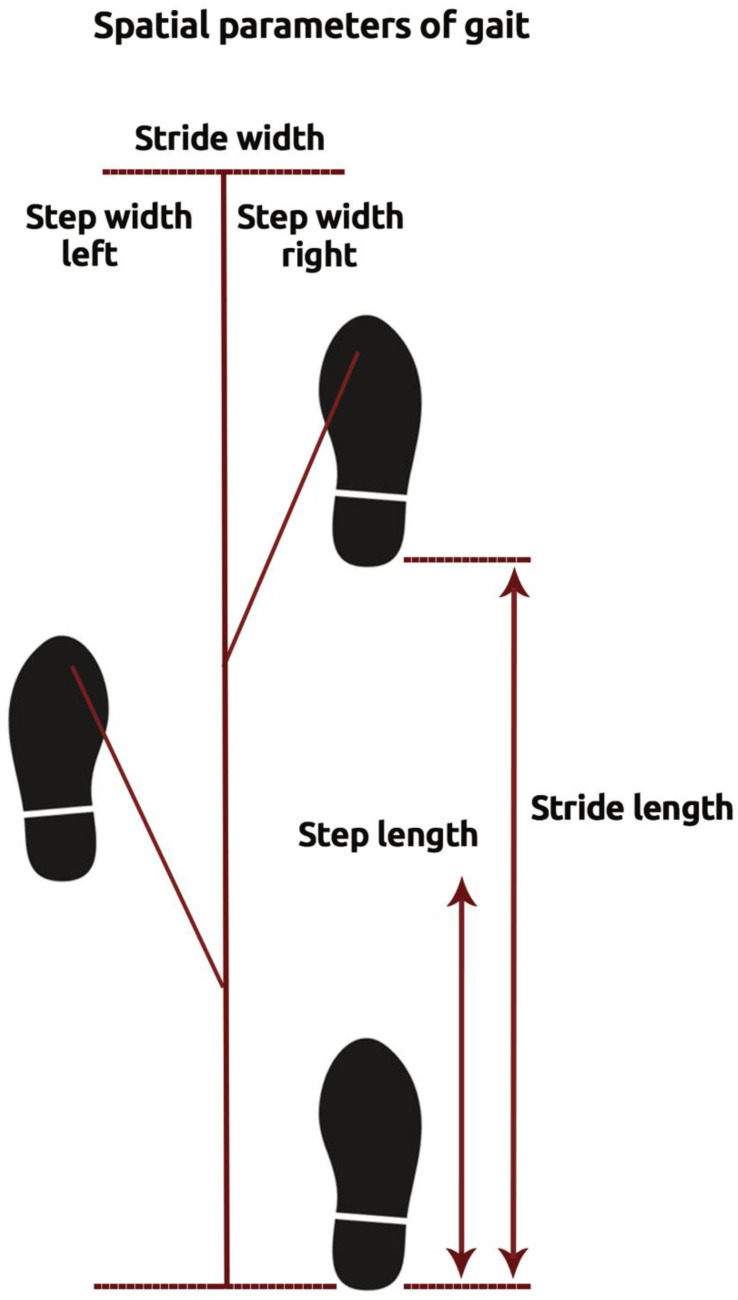
Illustration of spatial parameters of gait.

The primary endpoint was major adverse events (MAEs), defined as physical disability, falls, dependency, institutionalization, and mortality. Secondary endpoints were physical disability and mortality. Stride length was defined as the distance measured parallel to the line of progression, including two consecutive steps. Stride width was defined as side-to side distance between the heel of the current foot and heel of the next opposite foot. On the other hand, step width was determined as the distance between the outermost borders of two consecutive footprints (Figure 1). The standard deviation of the three variables (stride length) was used to represent the variability of the stride length [12]. Stride length was normalized to the subject’s height using the following formula [13]:

Normalized Stride Length  =  Stride Length (cm)/Height (cm)

All endpoints were evaluated at the longest available follow-up according to individual study protocols.

### 2.5. Data Extraction

Eligible studies were reviewed, and the following data were extracted: (1) first author’s name; (2) year of publication; (3) study design; (4) baseline of stride length and outcome; (5) patients’ baseline characteristics; (6) follow-up duration; (7) age and gender of participants.

### 2.6. Quality Assessment

Assessment of risk of bias and applicability concerns in the included studies was evaluated by the same investigators using Newcastle-Ottawa Scale (NOS) for cohort studies. Three domains were evaluated with the following items: (1) Selection, (2) Comparability, and (3) Exposure (assessment of outcome). The risk of bias in each study was judged to be “good”, “fair”, or “poor” [14].

### 2.7. Statistical Analysis

The meta-analysis was conducted using Statistical analysis, performed using the RevMan (Review Manager (RevMan) Version 5.1, The Cochrane Collaboration, Copenhagen, Denmark), with two-tailed *p* < 0.05 considered as significant. Weighted mean differences (WMDs) and 95% confidence interval (CI) are presented as summary statistics. Mean and standard deviation (SD) values were estimated using the method described by Hozo et al. [15]. Meta-analyses were performed with random effects models, as heterogeneity of effects among studies was expected. The generic inverse variance method was used to combine log Odds Ratio (log OR) and standard errors of the log OR (SElogORs). The log ORs were adjusted for a common set of co-founders across studies, such as age and gender. Heterogeneity between studies was assessed using the Cochrane Q test and *I*^2^ statistic. As a guide, *I*^2^ < 25% indicated low, 25–50% moderate, and >50% high heterogeneity [15]. To assess baseline cut-offs of stride length and stride length variability that could predict adverse clinical events, we performed hierarchical summary receiver operating characteristic (ROC) analysis using the Rutter and Gatsonis model [16]. Summary sensitivity and specificity with 95% CI for individual studies based on true positive (TP), true negative (TN), false positive (FP), and false negative (FN) were computed using the diagnostic random-effects model [17]. Potential publication bias was assessed using visual inspections of Begg’s funnel plot asymmetry and Egger’s weighted regression test.

## 3. Results

### 3.1. Search Results and Trial Flow

The preliminary screening ruled out articles whose titles and/or abstracts were not relevant. Two hundred and two studies were considered as potentially relevant, and after a stringent selection process, 11 articles met the inclusion criteria [18,19,20,21,22,23,24,25,26,27,28]. A listing of the study selection procedure and flow chart is shown in Figure S1.

### 3.2. Characteristics of Included Studies

Eleven cohort studies covering an older adult population of 14,167 were included in the analysis, with a mean follow up duration of 33.05 months. The subjects’ mean age was 75.4 ± 5.6 years, 55.8% were females, and the mean stride length adjusted to height was 0.79 ± 0.4 m for males and 0.71 ± 0.3 m for females. The main characteristics of the studies included in the meta-analysis are presented in Table 1.

### 3.3. Baseline Parameters of Gait in Individuals with and without Clinical Events

**Figure 2:**
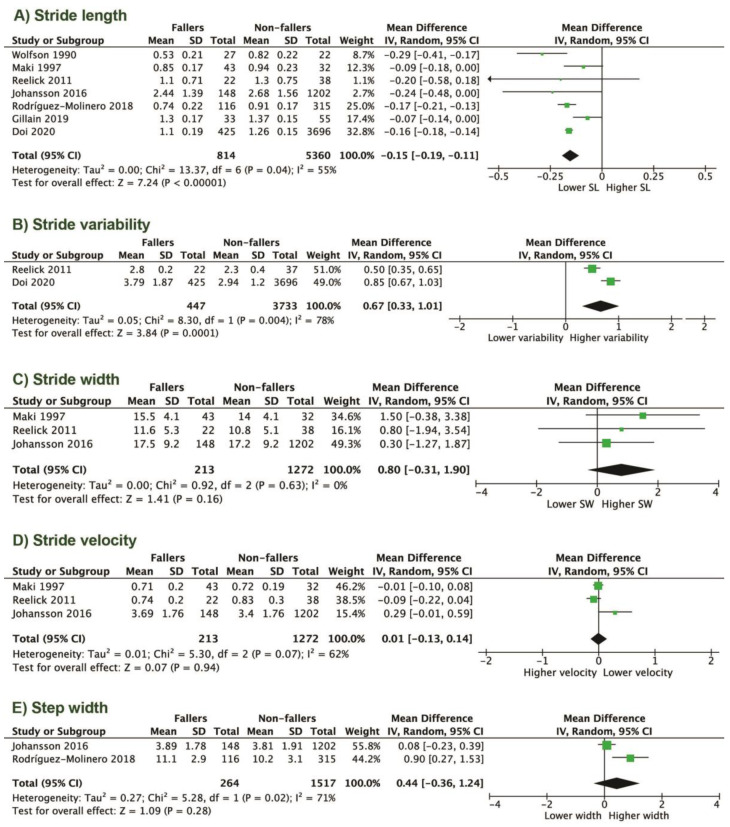
Gait parameters in fallers compared to non-fallers: (**A**) stride length; (**B**) stride variability; (**C**) stride width; (**D**) stride velocity; (**E**) step width.

Of the 14,167 studied individuals, falls occurred in 1383 (9.76%). Seven of eleven studies analyzed the stride length in participants with and without falls. Baseline stride length was shorter, with a weighted mean difference (WMD) of −0.15 (−0.19 to −0.11, *p* < 0.001), and stride length variability was higher, with a WMD of 0.67 (0.33 to 1.01, *p* < 0.001) in fallers compared to non-fallers (Figure 2A,B). Overall daily physical activities were lower in fallers compared to non-fallers (data from three cohorts): 61.8 vs. 71.6%; RR 0.69, (CI 0.56–0.84; *p* = 0.0007; Figure S2). Other gait parameters, including stride velocity WMD 0.01 (−0.13 to 1.14, *p* = 0.94), stride width WMD 0.80 (−0.31 to 1.90, *p* = 0.16), and step width WMD 0.44 (−0.36 to 1.24, *p* = 0.28), were not different between the two groups (Figure 2C–E). In a sub-analysis based on gender, the stride length was longer and walking speed slower in males (*p* < 0.001 and *p* = 0.04, respectively) compared to females (Figure S3).

### 3.4. Predictors of Adverse Clinical Outcomes

**Figure 3:**
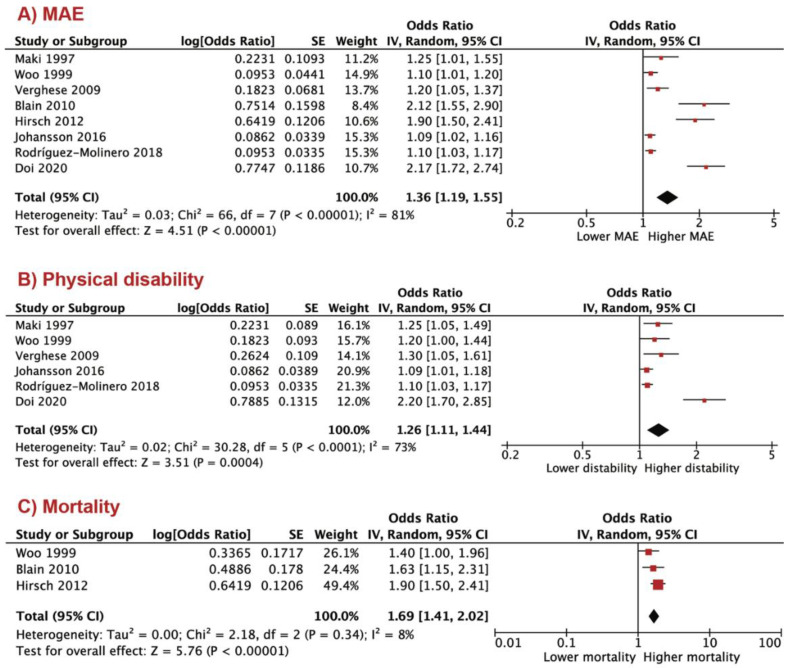
Shorter stride length in predicting adverse clinical events: (**A**) major adverse event (MAE); (**B**) physical disability; (**C**) mortality.

**Figure 4:**
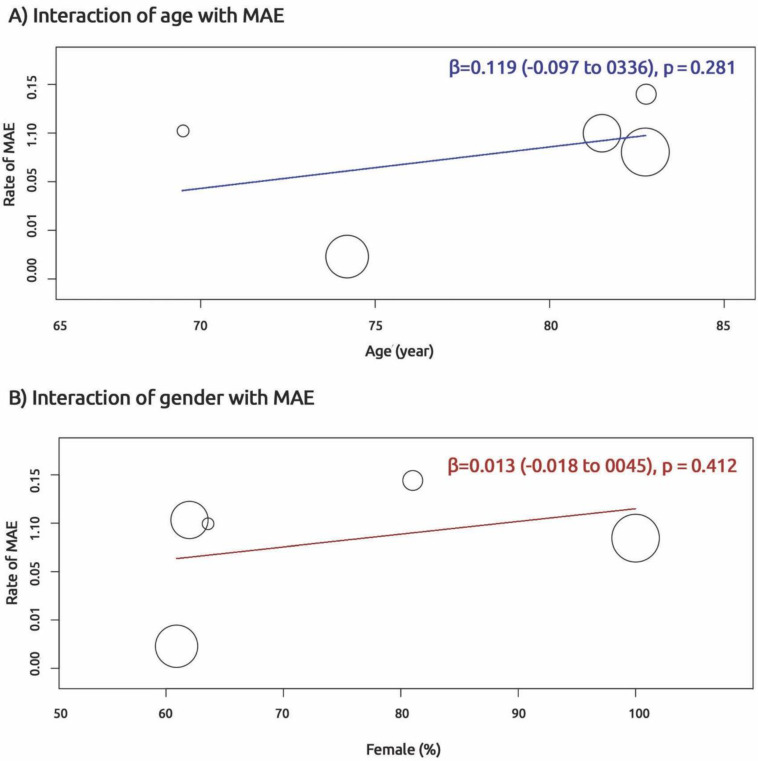
Interaction of age and gender with major adverse events (MAE).

At follow up, 3839 (27%) patients had clinical adverse events. The shorter stride length predicted MAE OR 1.36 (95% CI; 1.19 to 1.55, *p* < 0.001), physical disability OR 1.26 (95% CI; 1.11 to 1.44, *p* = 0.004) and mortality OR 1.69 (95% CI; 1.41 to 2.02, *p* < 0.001; Figure 3A–C). To test interaction between demographic indices and MAE, we performed a meta-regression analysis. No interaction was found between MAE and age, β = 0.119 (−0.097 to 0.336, *p* = 0.281) as well as MAE and female gender β = 0.013 (−0.018 to 0.045, *p* = 0.412; Figure 4).

**Figure 5:**
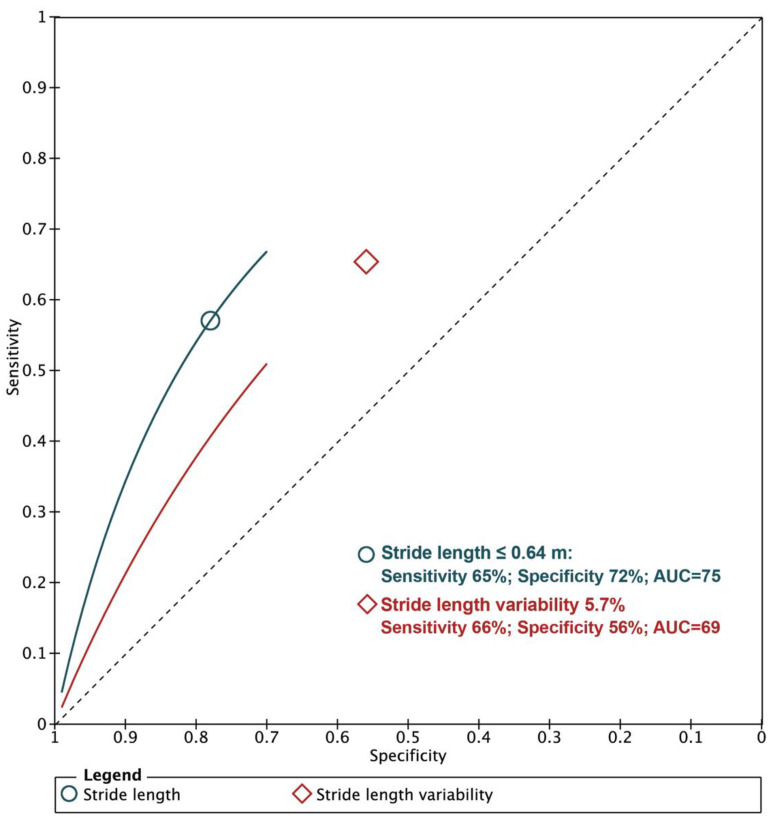
Diagnostic accuracy of stride length in predicting major adverse events (MAE).

A baseline normalized stride length ≤ 0.64 m was more accurate in predicting the combined adverse clinical events, with a summary sensitivity of 65% (58–71%), a specificity of 72% (69–75%) and an accuracy of 75.5% (74.2–76.7%) compared to a stride length variability of 5.7%, with a summary sensitivity of 66% (61–70%), a specificity of 56% (54–58%) and an accuracy of 57.1% (55.5–58.6%; Figure 5).

### 3.5. Risk of Bias Assessment

Eight papers (73%) had good quality, and the remaining 27% had fair quality (Table S1). There was no evidence for publication bias based on the Begg’s rank correlation test and Egger’s test.

## 4. Discussion

### 4.1. Findings

To our knowledge, the current meta-analysis is the first to evaluate the effects of stride length on clinical outcomes in older adults. The results of this meta-analysis of 11 studies with 14,167 participants revealed the following: (a) baseline stride length was shorter and stride length variability was higher in the older population who developed adverse clinical events compared to those with no clinical events, while the other gait parameters were not different between groups; (b) the shorter stride length predicted MAE, physical disability and mortality in the older adults; (c) a baseline stride length ≤ 0.64 m had higher accuracy in predicting adverse clinical events compared to a stride length variability of 5.7%.

### 4.2. Data Interpretation

Many researchers have focused on associations between gait speed, physical disability and other adverse events in older adults [29,30,31]. It has been reported that slowing the walking speed reflects health and functional status and predicts survival [32,33]. However, gait is a complex neuromotor behavior, with many measurable facets in addition to velocity. It also has an intricate relationship with different aspects of the psychomotor system. In addition, other quantitative parameters of gait, such as swing phase, stride length and gait variability, demonstrated better predictive value of disability compared to speed alone [31,34]. Body balance is a crucial factor in maintaining healthy and safe walking and for avoiding falls. Shorter stride length and higher stride length variability are two important factors directly involved in the mechanisms of poor balance, which has been shown as a marker of low survival, physical disability and other adverse clinical events [34,35]. These parameters might indicate a certain body inability to improve or recover from future adverse events. Our results support this concept and strengthen further the importance of shorter stride length as a robust marker in predicting MAE, physical disability and mortality. In addition, our analysis proposes a summary cut-off value for stride length of 0.64 m with high accuracy compared with stride length variability in predicting clinical events; our suggestion for future direction is to establish cut-off values based on the demographic characteristics of each country.

### 4.3. Clinical Implications

Stride length and stride length variability, as important parameters of body balance during walking in older adults, can be a target for intervention through medical, rehabilitative and health-promoting behavioral strategies. These interventions should aim at maintaining long strides in order to sustain improved long-term physical function and survival in older adults.

### 4.4. Limitations

The most significant limitations of this meta-analysis are related to the limited number of available publications, although the population number in each study was satisfactory. We would have liked to report the different cut-offs of stride length according to different countries and different ethnicities, but these data were not consistently available in all included studies. We did not have control over various measurements of subjects’ gait but had no reason to doubt the reliability of the previously published data. The available data do not allow us to draw conclusions about the importance of medical intervention in stride length. Future studies may be required to determine the impact of intervention through medical, rehabilitation and health-promoting behavioral strategies on better clinical outcomes.

## 5. Conclusions

The results of this meta-analysis support the significant value of stride length in predicting life-threatening clinical events in older adults. A stride length of 0.64 m accurately predicts the occurrence of future clinical events and thus should provide potential guidance towards optimum individual exercise and support.

## Figures and Tables

**Table 1:** Main characteristics of studies included in the study.

Study	Study	Country	Sample	Adverse	Inclusion	Exclusion	Clinical	Follow-up
(Year)	Design		Size	Events	Criteria	Criteria	Outcomes	(Months)
Wolfson 1990	Cohort	USA	49	27	Elderly	Unstable due to	Physical	24
	(prospective)				population	dementia, terminal	disability	
	study					illness, behavioral		
						or neurological		
						problems		
Maki 1997	Observational	USA	75	43	Elderly	Elderly population	MAE,	12
	(prospective)				population	that failed to	physical	
	study				able to	meet these	disability	
					walk 10 m,	criteria		
					to understand			
					verbal cues			
Woo 1999	Cohort	China	2032	1215	Elderly	NR	MAE,	36
	(prospective)				population		mortality	
	study				able to walk			
					unaided			
Verghese 2009	Cohort	USA	597	226	Aged ≥ 70	Audiovisual loss,	Physical	20
	(prospective)				years	bed bound	disability	
	study					due to illness,		
						institutionalization		
Blain 2010	Cohort	France	1300	410	Aged ≥ 75	Bilateral hip	Mortality	96
	(prospective)				able to	replacement,		
	study				walk indepen-	previous		
					dently and having	hip fracture		
					cognitive			
					health			
Reelick 2011	Cohort	Netherlands	60	38	Elderly	Insufficient vision,	MAE,	6
	(prospective)				population	MMS Examination	physical	
	study				able to	score < 15,	disability	
					walk 15 m	neurological		
					independently	disfunction		
Hirsch 2012	Cohort	USA	4182	1901	Elderly	Wheelchair bound	MAE,	24
	(prospective)				population	or receiving		
	study				aged ≥ 75	hospice treatment,	mortality	
	study					radiotherapy,		
						chemotherapy		
Johansson	Observational	Sweden	1350	148	Elderly	No eligible	MAE,	12
2016	(prospective)				population	participant	physical	
	study				age of exactly	was excluded	disability	
					70 years			
Rodríguez-	Cohort	Spain	431	116	Elderly	Participants who	MAE,	60
Molinero 2018	(prospective)				population	were unable	physical	
	study				aged ≥ 65 years	to walk	disability	
						autonomously		
Gillain 2019	Cohort	France	105	35	Elderly	History of falls,	MAE	24
	(prospective)				aged ≥ 65 years	gait disorders,		
	study				living	Parkinson’s disease,		
					independently	hip or knee		
					at home	Prosthesis, etc.		
Doi 2020	Cohort	Japan	4121	425	Elderly	Having any	MAE,	49.6
	(prospective)				population	dependency, ADL,	physical	
	study				aged ≥ 65 years	stroke, Parkinson’s,	disability	
						disease, etc.		

Abbreviations: MAE: major adverse events; ADL; Activities of Daily Living; m: meter.

## Data Availability

Meta-analysis.

## Supplementary Materials

The following are available online at https://www.mdpi.com/article/10.3390/jcm10122670/s1, Figure S1: Flow chart, Figure S2: Figure S3: Physical activity in fallers compared to non-fallers, Table S1: Assessment of risk of bias in the included studies using Newcastle-Ottawa Quality Assessment Scale (NOS) for cohort (observational) studies.
