# Supplementary material for: Stride Length Predicts Adverse Clinical Events in Older Adults: A Systematic Review and Meta-Analysis
Source: J Clin Med. 2021 Jun 17;10(12):2670. doi: 10.3390/jcm10122670 (PMC8235531; doi:10.3390/jcm10122670)
Supplement: Supplementary file 1 [file jcm-10-02670-s001.zip › jcm-1210226-supplementary.pdf]

Supplementary data

Figure S1. Flow chart

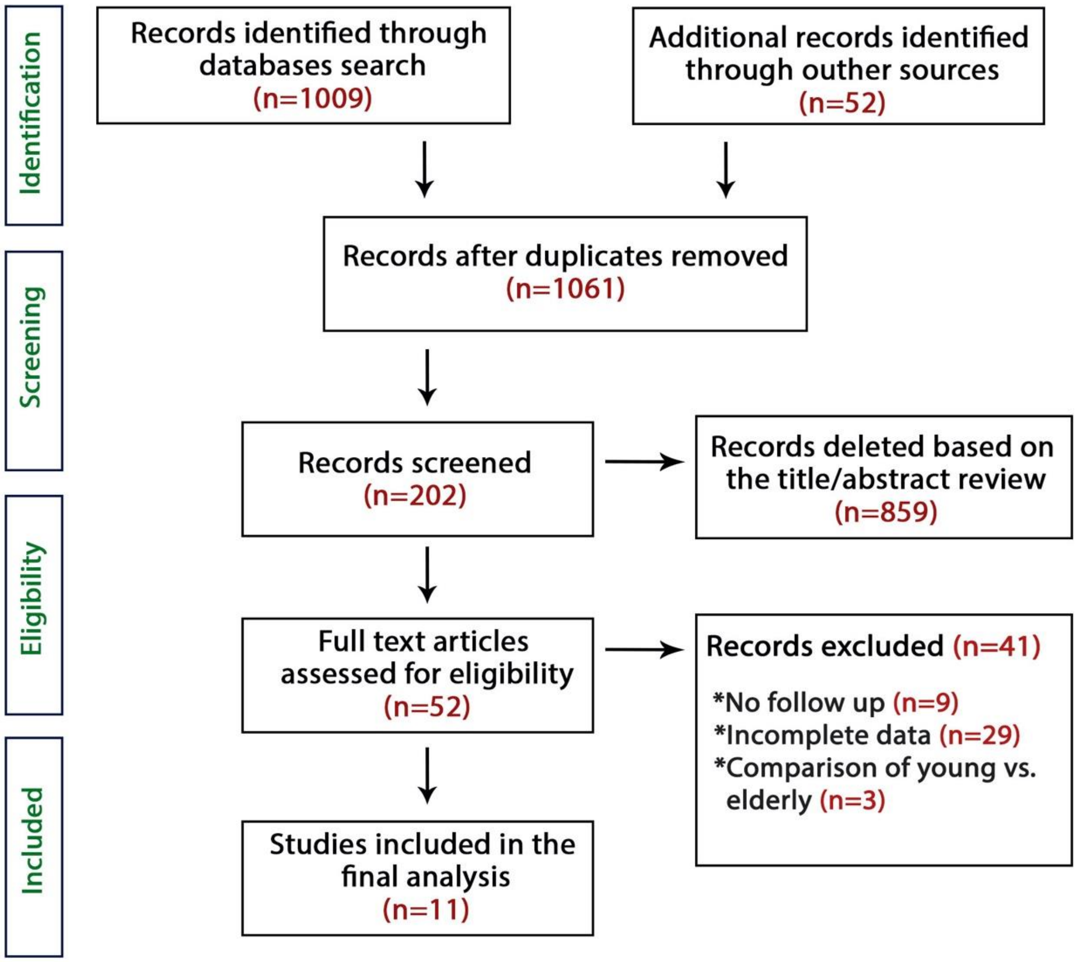

Figure S2. Gait parameters in female compared to male

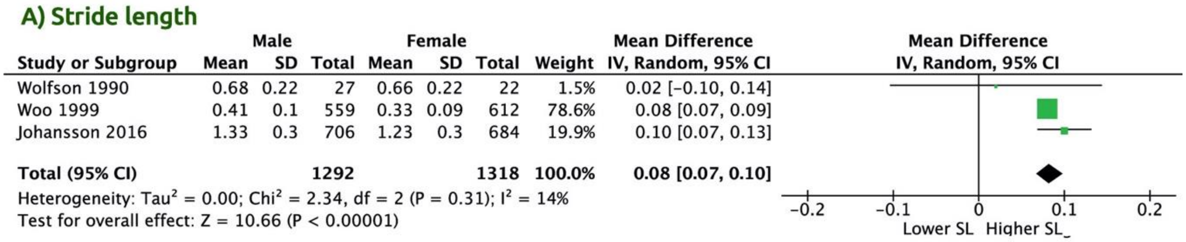

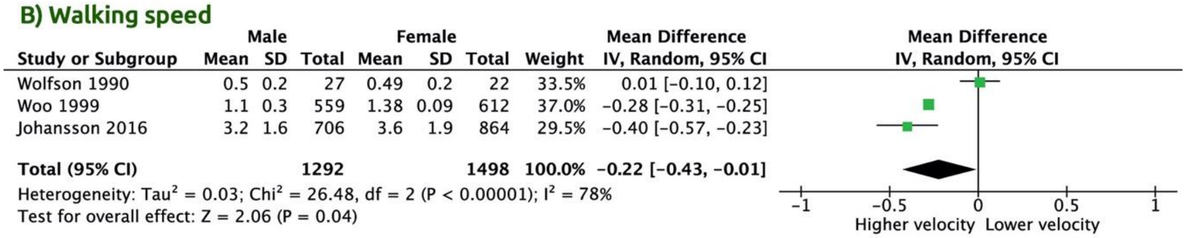

Figure S3. Physical activity in fallers compared to non-fallers

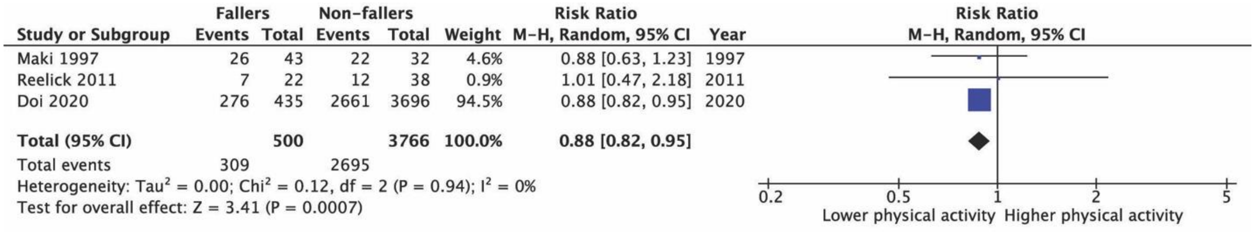

Table S1. Assessment of risk of bias in the included studies using Newcastle-Ottawa Quality Assessment Scale (NOS) for cohort (observational) studies.

**Study, year**	**Selectio 1**	**Selectio 2**	**Selectio 3**	**Selection 4**	**Comparabilit 1**	**Exposur 1**	**Exposure 2**	**Exposur 3**	**Quality**
Wolfson 1990	a)	a)	b)	b)	b)	b)	a)	b)	Fair
Maki 1997	a)	a)	a)	c)	b)	a)	b)	b)	Good
Woo 1999	a)	b)	b)	b)	b)	a)	b)	b)	Fair
Verghese 2009	a)	a)	b)	b)	b)	b)	a)	b)	Good
Blain 2010	a)	b)	a)	c)	a)	a)	b)	b)	Good
Reelick 2011	b)	b)	b)	b)	a)	b)	b)	b)	Fair
Hirsch 2012	a)	a)	a)	c)	a)	a)	b)	a)	Good
Johansson 2016	a)	a)	b)	b)	a)	a)	b)	a)	Good
Rodríguez-Molinero [illegible]	a)	a)	a)	b)	a)	a)	b)	a)	Good
Gillain 2019	a)	b)	b)	b)	a)	a)	b)	a)	Good
Doi 2020	a)	a)	b)	b)	a)	a)	b)	a)	Good

Legend: NOS: Selection- 1: a), b) one star, c), d) no star; Selection- 2: a) one star, b, c) no star; Selection- 3: a), b) one star, c), d), e) no star, Selection- 4: a) one star, b) no star; Comparability: a), b) one star, c) no star; Exposure-1: a),b) one star, c), d), e) no star; Exposure-2: a) one star, b) no star; Exposure-2**:** a),b) one star, c), d), no star.
Good quality: 3 or 4 stars in selection domain AND 1 or 2 stars in comparability domain AND 2 or 3 stars in outcome/exposure domain
Fair quality: 2 stars in selection domain AND 1 or 2 stars in comparability domain AND 2 or 3 stars in outcome/exposure domain
Poor quality: 0 or 1 star in selection domain OR 0 stars in comparability domain OR 0 or 1 stars in outcome/exposure domain
